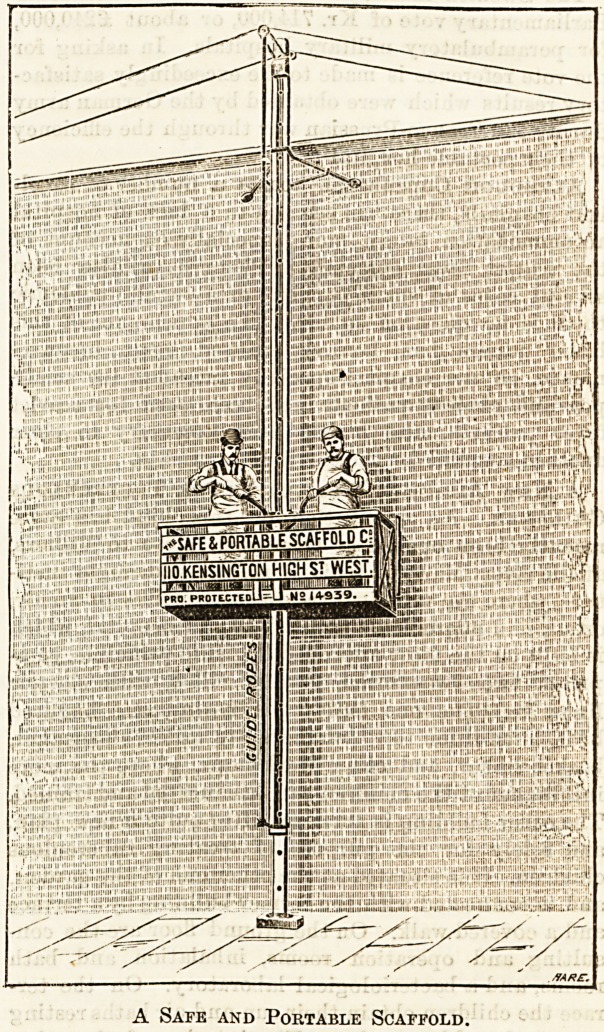# Practical Departments

**Published:** 1894-04-07

**Authors:** 


### PRACTICAL DEPARTMENTS.

### A SAFE AND PORTABLE SCAFFOLD.

Many accidents are reported yearly, caused by falls from scaffolding insecurely erected or too slightly built, and the desirability of the general adoption of some such safety scaffold as the one illustrated below has been terribly emphasized within the last few days by the sad accident in Regent Street, where the slipping of a rope suspending a swing boat, on which some workmen were engaged in painting, has resulted in the loss of two lives, and in more or less serious injury to three or four people. The pole and platform scaffold is much to be recommended as a life-saving apparatus, being really absolutely safe, and allowing men working upon it to carry on their work of painting, repairing, &c., with ease, comfort, and consequent rapidity, thus ensuring saving of time in addition to other advantages. It is easily fixed, and can be moved in any direction by the men working on it without difficulty. It should be mentioned that any width of building may be covered by one fixing, and no further attention or trouble is required, as we have said, the workmen being able to raise or lower the scaffold or move it from side to side quite easily. Our illustration is given by kind permission of the Safe and Portable Scaffold Company, 110, Kensington High Street, from whom these scaffolds may be hired on moderate terms, and from a humanitarian point of view they ought certainly to be widely adopted.

A Safe and Portable Scaffold.

## Figures and Tables

**Figure f1:**